# A Tandem Chemical Vapor Deposition Platform for the Solvent‐Free Synthesis of Polypeptide Architectures

**DOI:** 10.1002/chem.202503611

**Published:** 2026-01-19

**Authors:** Domenic Kratzer, Martina Plank, Meike Koenig, Tahereh Mohammadi Hafshejani, Joerg Lahann

**Affiliations:** ^1^ Institute of Functional Interfaces (IFG) Karlsruhe Institute of Technology (KIT) Karlsruhe Germany; ^2^ Soft Matter Synthesis Laboratory Institute for Biological Interfaces 3 (IBG‐3) Karlsruhe Institute of Technology (KIT) Karlsruhe Germany; ^3^ Biointerfaces Institutes University of Michigan Ann Arbor Michigan USA

**Keywords:** biomaterials, end‐attached polypeptides, NCAs, solvent‐free approach, tandem CVD

## Abstract

The precise engineering of surfaces decorated with polypeptides is critical for advanced diagnostics, biomedical coatings, and cellular interfaces. However, conventional methods are plagued by the need for solvents, multistep procedures, substrate limitations, and the abundance of side reactions. Here, we report that two‐step chemical vapor polymerization can result in the fast, efficient, and substrate‐independent synthesis of polypeptide films without the use of solvents or excipients. The first step involves deposition of an initiator layer, i.e., poly(4‐amino‐*p*‐xylylene), via chemical vapor deposition (CVD) polymerization of 4,16‐diamino[2.2]paracyclophane. The second step involves evaporation and ring‐opening polymerization of *N*‐carboxy anhydrides. This fully integrated CVD approach ensures substrate‐independent, conformal growth of poly(propargyl‐(*S*)‐glycine) and poly(*O*‐propargyl‐(*S*)‐tyrosine) films of up to 198 nm thickness. The use of CVD processes eliminates the concern of side reactions, such as transfer and termination reactions, and is a prerequisite for the successful peptide micropatterning, demonstrated in this study. Successful peptide growth and post‐polymerization modifications via click chemistry were confirmed by time‐of‐flight secondary mass spectrometry, x‐ray photoelectron spectroscopy, and infrared spectroscopy. The application of entirely solvent‐free workflows to develop biomacromolecular coatings, such as the polypeptide films demonstrated in this study, addresses a critical gap in the pursuit of advanced and scalable biologization methods.

## Graphical abstract

A solvent‐free, two‐step chemical vapor polymerization enables efficient, substrate‐independent synthesis of surface‐attached polypeptide films. Initiator deposition via CVD is followed by NCA ring‐opening polymerization. Characterization confirms film formation and functionalization, allowing precise micropatterning and bioactive surface design for biomedical and industrial applications.

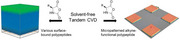

## 1 Introduction

The decoration of materials with interfaces that offer controlled chemical functionality and topography is a critical prerequisite for many biomedical applications. In recent years, there has been increasing interest in surface‐bound polypeptides, mainly due to their extensive potential for applications in biotechnology [1], including biosensors [2], chiral separation membranes [3], cell culture substrates [4], optical devices, and liquid crystal displays [5, 6, 7, 8]. Polypeptides possess the ability to exhibit distinct biological functions, such as conformational transition [9, 10] or permeability [11], and can serve as templates for the development of biomacromolecular architectures [7, 12, 13]. Modifying the interfacial properties of inorganic materials with synthetic polypeptides requires advanced and scalable processing technologies that must operate under conditions compatible with both, the biomolecular component (typically aqueous) and the abiotic components (typically organic solvents).

The preparation of synthetic polypeptides is generally limited to three fundamentally different strategies, i.e., solid‐phase peptide synthesis (SPPS), recombinant peptide synthesis, and ring‐opening polymerization (ROP) of *N*‐carboxy anhydrides (NCAs) [14, 15, 16]. In SPPS, a peptide anchored to a solid phase is assembled by successive addition and deprotection of protected amino acids, which can be labor‐intensive, tends to suffer from low efficiencies, and hence favors peptides with relatively low molecular weights [17, 18]. Recombinant peptide synthesis is versatile, but involves genetically modified organisms, such as bacteria or yeast, requires substantial post‐processing purification, and is thus associated with considerable time, effort, and cost [19, 20, 21]. Alternatively, ROP of NCA derivatives has been used to prepare a wide range of polypeptides [16, 22, 23]. Nucleophiles act as initiators, as they readily react with the NCA building blocks, releasing carbon dioxide as the only by‐product, which is a gas and rapidly dissipates. Compared to the methods mentioned above, ROP of NCAs offers versatility, simplified purification requirements, and a relatively simple and cost‐efficient implementation, but is plagued by the abundance of side reactions, such as chain transfer reactions and termination reactions [24].

In a further refinement of this approach, surface‐initiated vacuum deposition of NCAs has been demonstrated by Chang and Frank [25, 26]. In this approach, NCAs were first evaporated in a vacuum to react with surface‐bound initiators. High reaction efficiencies and increased process controllability were reported [25, 26, 27], but the process was, by and large, limited to silicon substrates [26, 27, 28, 29, 30, 31, 32, 33], and mostly solvent‐based processing was used to introduce initiator groups prior to vapor‐based ROP [34, 35]. Past efforts with vapor‐based polymerization of NCAs were hampered by the lack of micropatterning and post‐polymerization modification [6, 26, 29, 33].

Chemical vapor deposition (CVD) polymerization of functionalized [2.2]paracyclophanes is a solvent‐free surface functionalization method that delivers homogenous and high‐density reactive polymer coatings on a broad variety of materials and can be applied to substrates with complex geometries [36, 37, 38, 39, 40, 41]. Members of the poly‐*p*‐xylylene family (PPX, trade name: Parylene) are already commercially established and are utilized as a barrier coating for implants, stents, pacemakers, or catheters [42, 43, 44]. In past publications, nucleophilic amine groups have been immobilized via CVD polymerization for the immobilization of biomolecules [45], or for the use as stimuli‐responsive coatings [46]. Here, CVD polymerization constitutes a critical step of the tandem CVD approach because it introduces a dense and homogenous layer of primary amine groups for subsequent initiation of the ROP of NCAs [47].

Due to the above‐discussed technical limitations, and the fact that published work has been limited to a limited range of NCAs, typically derived from natural amino acids, these types of peptide coatings have not yet lived up to their full potential, if it comes to biomedical coatings. Our work seeks to address these shortcomings by taking advantage of recent progress in the synthesis of NCAs [48]. Prior to this work, strategies for the synthesis of NCAs involved the cumbersome phosgenation of amino acids under dry and inert conditions, which required the use of a glove box to conduct the workup [23, 49]. The addition of an effective acid scavenger, which prevents degradation of the NCA during the reaction, effectively addresses these limitations. Importantly, this methodology can be extended to monomers with side groups that react orthogonally to the ROP, simplifying post‐polymerization modifications.

**SCHEME 1:**
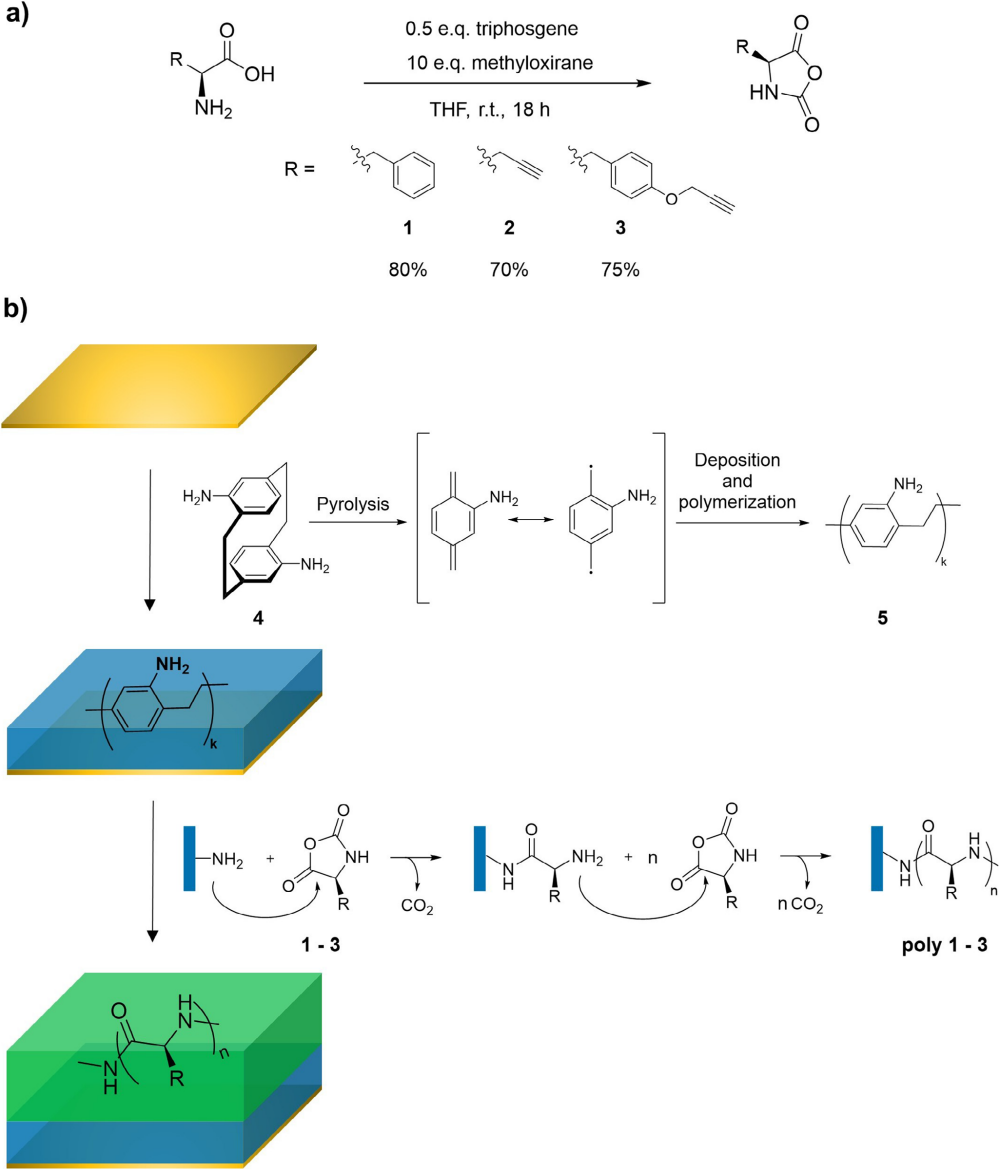
Synthesis of the NCA monomers (a) and schematic representation of the two‐step CVD process toward substrate‐bound homopolypeptides (b). Amino‐functionalized initiator coatings were prepared by CVD of 4,16‐diamino[2.2]paracyclophane (**4**), resulting in a homogenous and stable PPX film (**5**) on the substrate. The surface‐bound amino groups serve as an initiator for the vapor‐based ROP of phenylalanine‐NCA (**1**), propargylglycine‐NCA (**2**), and propargyltyrosine‐NCA (**3**).

Herein, we report a solvent‐free, tandem CVD approach, which is an inherently substrate‐independent process offering the potential to implement micropatterning techniques [34, 35, 50, 51, 52]. Using the scavenger method, we synthesized literature‐unknown propargyltyrosine‐NCA films with excellent solvent stability. The solvent‐free, high‐vacuum conditions explored in this approach were selected to maintain the conditions necessary for the living polymerization of NCAs with primary amines, thereby minimizing side reactions, such as chain transfer and premature termination [53, 54, 55]. Therefore, this work extends the scope of vapor‐based peptide syntheses, with respect to both available NCA derivatives and substrates [53]. More conceptually, we developed a tandem CVD approach for the preparation of surface‐bound polypeptides, starting with the synthesis of NCA monomers, followed by a two‐step surface modification and the subsequent characterization of the resulting polypeptide surfaces (Scheme 1).

## 2 Results and Discussion

**FIGURE 1:**
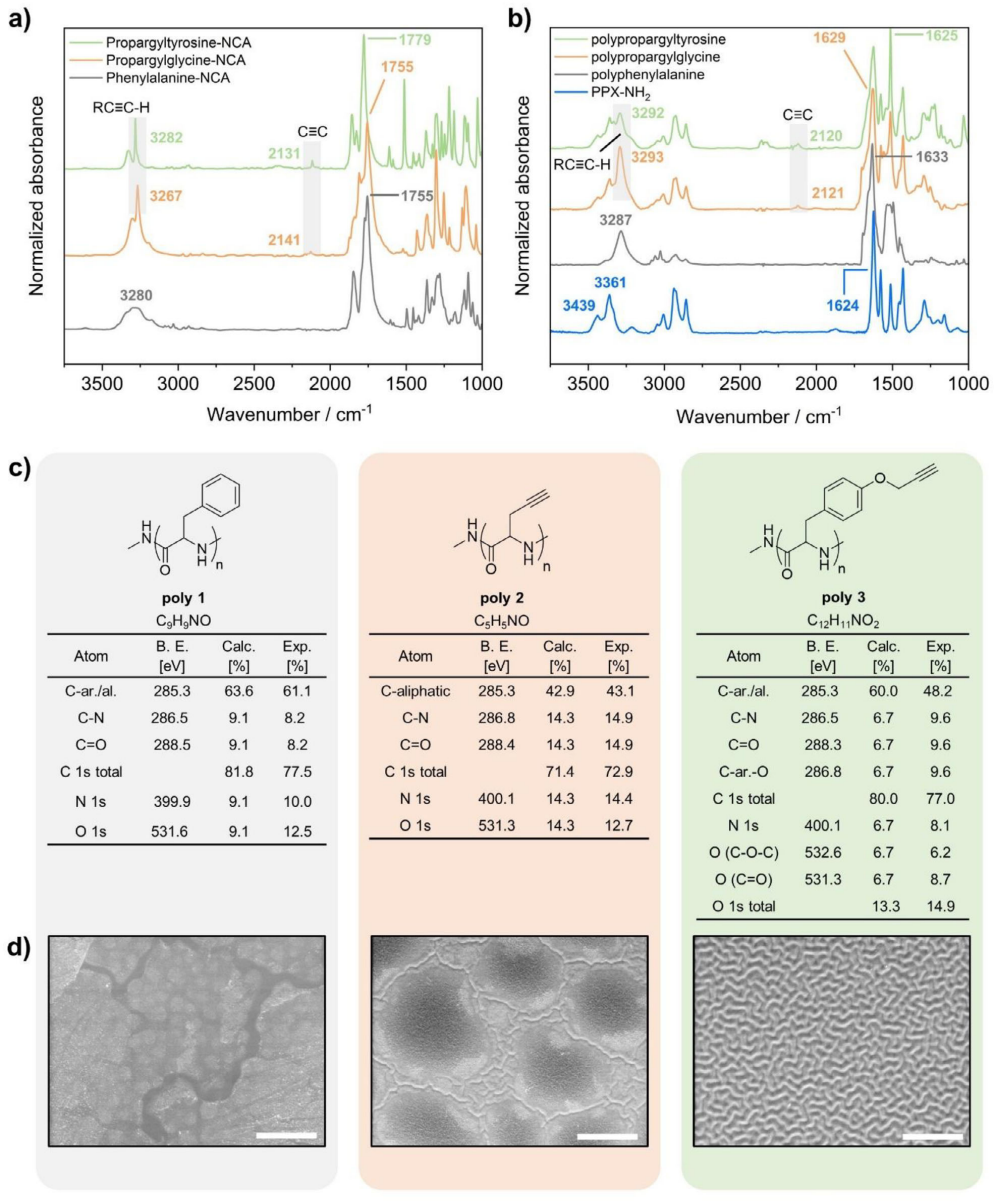
Characterization of NCAs and their corresponding surface‐bound polypeptide films. ATR‐FTIR spectra of the synthesized NCA monomers (a) and polypeptide films on initiator‐coated surfaces (b). (c) Tables showing the chemical composition of **poly 1** (left), **poly 2** (middle), and **poly 3** (right) on substrates, determined by XPS, and (d) their corresponding topographic SEM images by channel mixing of SE and BSE signals (scale bar: 5 µm).

NCA monomers were first synthesized according to a procedure recently published by Tian et al. with slide modifications (Scheme 1a) [48]. Literature known (*S*)‐phenylalanine‐NCA (**1**) and the alkyne‐functional monomers (*S*)‐propargylglycine‐NCA (2), as well as literature unknown *O*‐propargyl‐(*S*)‐tyrosine‐NCA (3), were synthesized via ring‐closure of the corresponding amino acids with triphosgene. Critical to this approach, methyloxirane was employed as an effective scavenger of hydrochloric acid, thereby preventing acid‐catalyzed degradation of the formed NCAs. The NCA monomers were isolated in excellent yields between 70% and 80% and with high purity. The tandem CVD approach used to deposit the peptide film is illustrated in Scheme 1b. First, substrates were coated by CVD polymerization of precursor **4**, resulting in initiator films with a polymer thickness ranging from 90 to 140 nm, as confirmed by spectroscopic ellipsometry. The amino‐functionalized polymer films had a chemical composition consistent with published results [56, 57], as confirmed by IR spectroscopy (Figure 1b, blue line) and XPS (Supporting Information‐Figure 1a).

**FIGURE 2:**
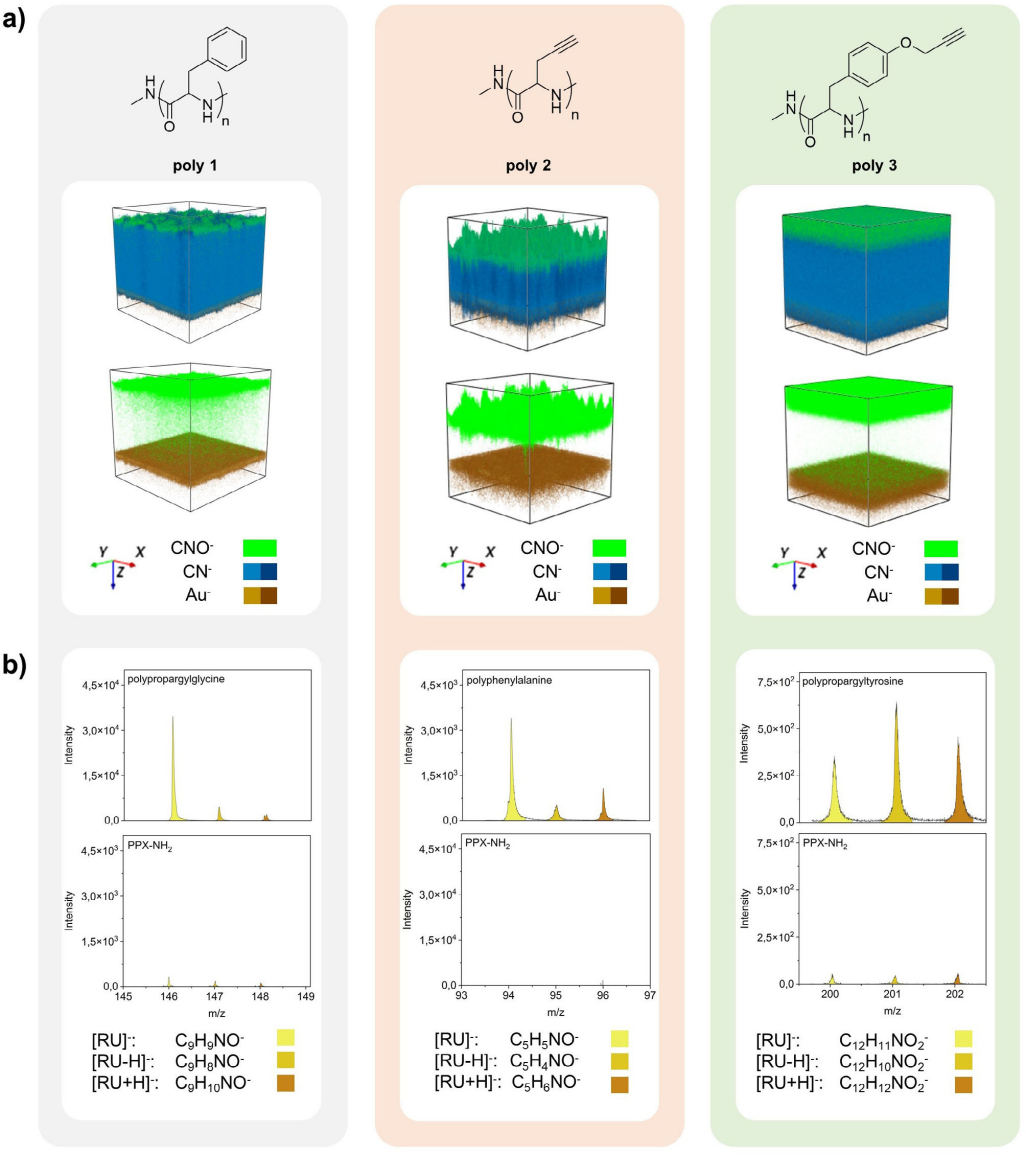
Analysis of the multicomponent system using ToF‐SIMS. (a) Depth profiles displaying the 3D main distribution of detected CNO^−^, CN^−^, and Au^−^ ions (*x* and *y* 300 µm, *z* not to scale. To each type of ion was assigned a specific color code, with CN^−^ and Au^−^ each represented by a minimum and maximum color intensity.) of **poly 1** (left), **poly 2** (middle) and **poly 3** (right) on gold substrates. (b) Surface spectra showing characteristic mass peaks, like the fragment of the repeating unit (RU^−^) and its neighboring fragments ([RU+H]^−^, [RU−H]^−^) of **poly 1** (left), **poly 2** (middle), and **poly 3** (right) in comparison with their corresponding PPX‐NH_2_ initiator layer (bottom).

The second step involved vapor‐based ROP of NCA derivatives **1**, **2**, or **3**. Here, effective ROP was observed for reduced pressures (10^−4^ mbar) and elevated deposition temperatures (60°C–80°C). Under those conditions, the various NCA's evaporated with minimal decomposition and were effectively transferred into the reaction one, where they readily reacted with the initiator layer of the substrate. To optimize the reaction conditions and validate the custom‐built setup (Supporting Information‐Figure 2), test reactions were performed using the literature‐known NCA **1** [26, 27, 28]. At a deposition temperature of 60°C, **poly 1** films with thicknesses between 15 and 45 nm were obtained, depending on the amount of starting material. These findings suggest that the ROP of NCA's can proceed at significantly lower deposition temperatures (60°C) than what was previously reported (100°C), potentially reducing the likelihood of side reactions, as observed in solution‐based polymerizations [27, 58, 59].

Following the successful tandem CVD of NCA **1**, experiments were extended to the alkyne‐functional NCAs **2** and **3**. Aliphatic NCAs such as alanine‐NCA or valine‐NCA are known for their thermal instability and can undergo thermal polymerization even at temperatures below 0°C [26, 60]. For the aliphatic NCA **2**, a lower evaporation temperature of 110°C was deemed optimal for evaporation and to minimize side reactions, such as thermally induced polymerization. The optimum deposition temperature was found to be 60°C, and the reaction time was set to be 10 min. NCA quantities ranging from 25 to 45 mg of starting material consistently produced **poly 2** films with a thickness of approximately 25 nm. For comparison, an NCA amount below 20 mg resulted in inhomogeneous films, with the polypeptide appearing only on islands scattered across the entire surface.

**SCHEME 2:**
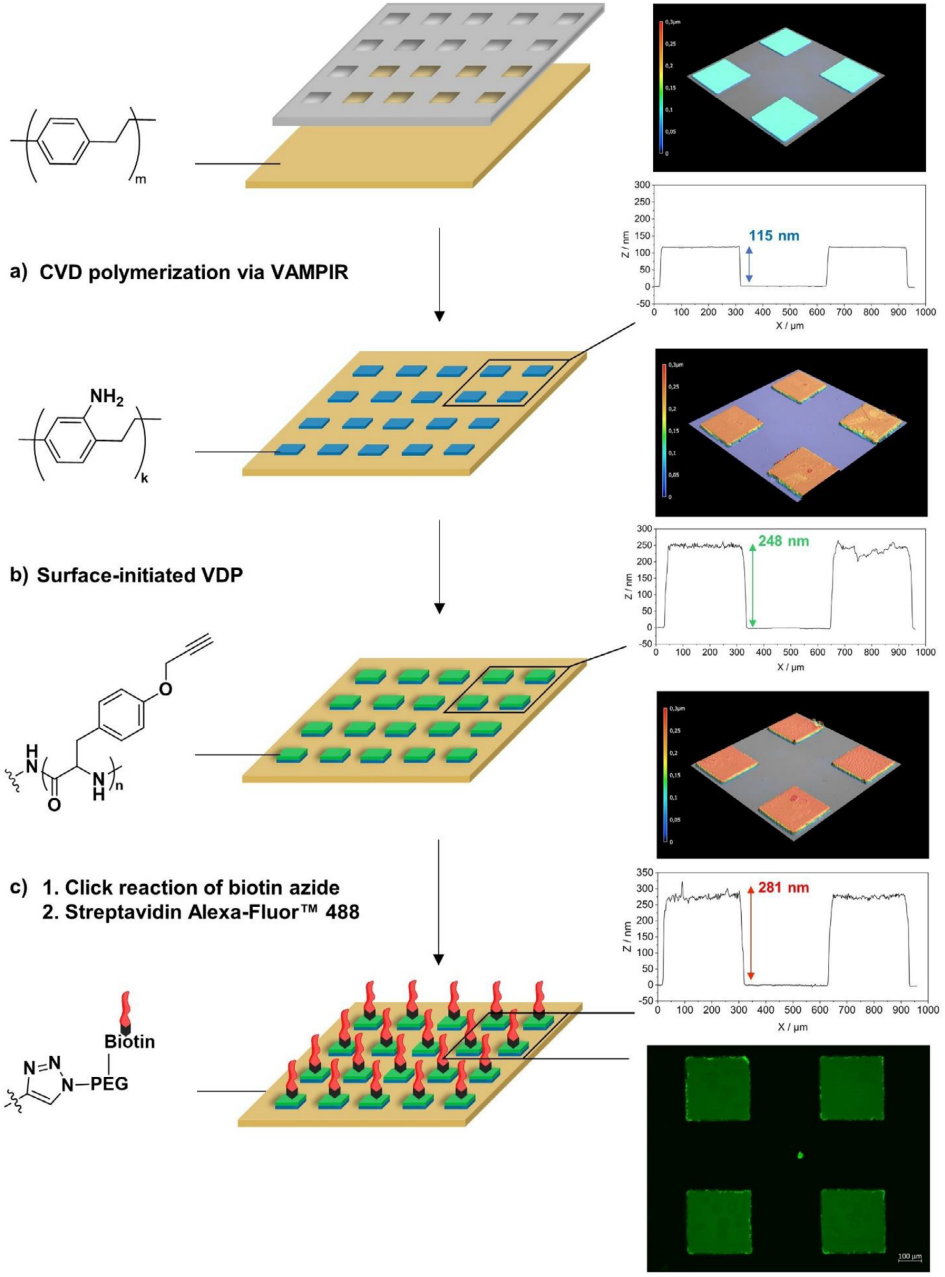
Schematic diagram illustrating the immobilization of streptavidin Alexa Fluor 488 on a micro‐structured polypropargyltyrosine surface prepared via the VAMPIR technique. The patterned surface was prepared in three steps and was investigated by white light interferometry (3D‐image and height profile) and fluorescence microscopy (c), showing (a) a micro‐structured PPX‐NH_2_ initiator layer with a thickness of 115 nm on a substrate pre‐coated with nonfunctional PPX after removal of the PDMS micro stencil. (b) Micropattern after selective surface‐initiated vapor deposition polymerization of propargyltyrosine‐NCA, resulting in a structured polypeptide surface with a height difference of 248 nm. (c) Final patterned surface after the attachment of biotin‐dPEG7‐azide and subsequent binding of streptavidin Alexa Fluor 488 conjugate (top) and the corresponding fluorescence micrograph showing fluorescence only within the squares (bottom).

**FIGURE 3:**
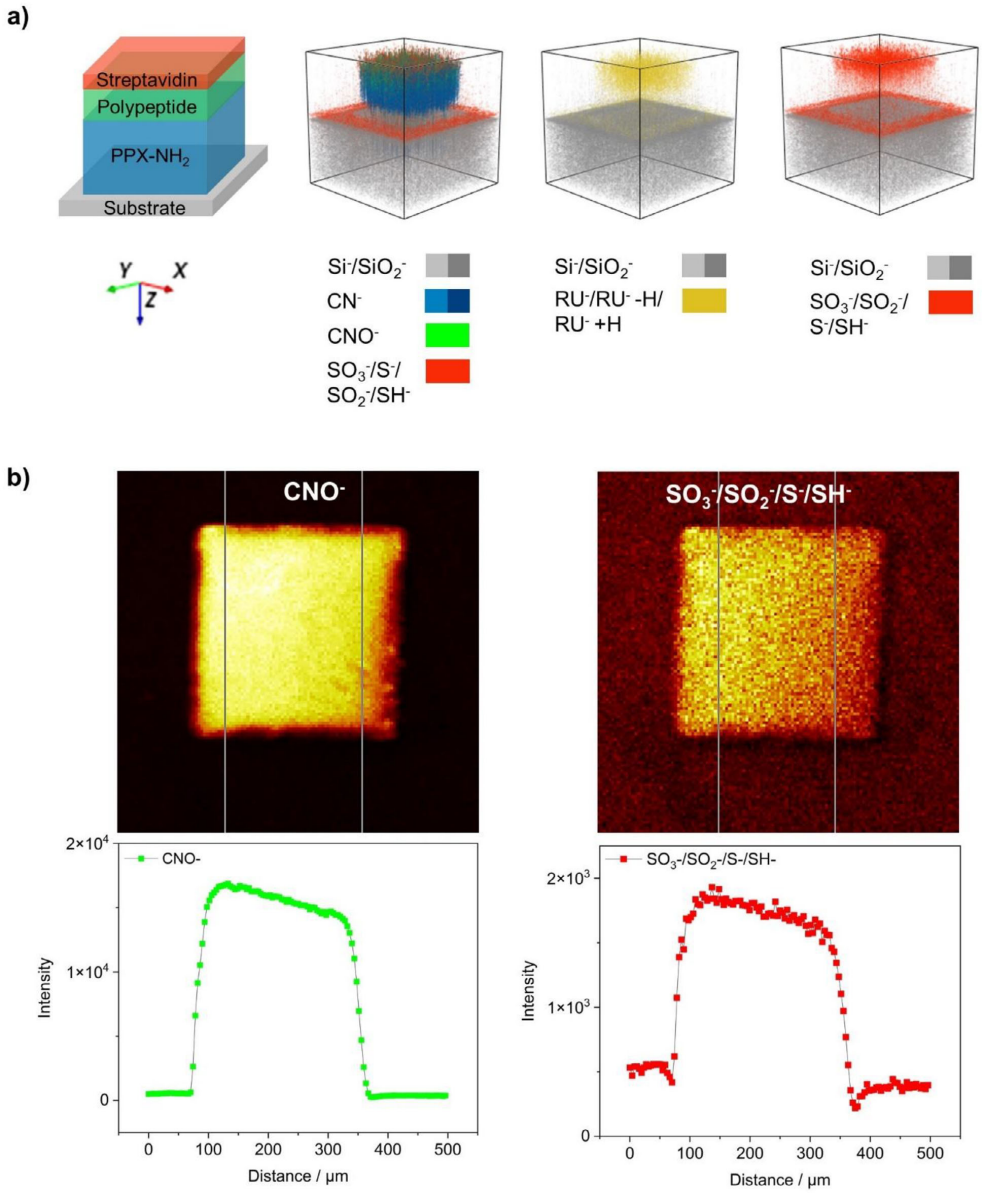
In‐depth analysis of micropatterns functionalized with **poly 3** for the selective immobilization of streptavidin Alexa Fluor 488. (a) ToF‐SIMS depth profiles of micro‐structured **poly 3** films on silicon substrate displaying a selection of characteristic polymer fragments, depicted as 3D ion main distribution (*x* and *y* 300 µm, *z* not to scale). (b) Line scan plot of the ToF‐SIMS depth profile using the line type *y*‐area depicted in the intensity image of the related measurement, indicating the selective functionalization by polymerization of propargyltyrosine NCA shown by the CNO^−^ signal, as well as the selective subsequent adsorption of streptavidin Alexa Fluor 488, illustrated by SO_3_‐/SO_2_‐/S‐/SH^−^ ‐fragments.

Due to its higher thermal stability, ROP of the aromatic NCA **3** proceeded without complications, making it the best candidate for further post‐polymerization modifications. Reproducible and homogeneous polypeptide layers were achieved at an evaporation temperature of 142°C (m.p. = 141°C) and a deposition temperature of 80°C. Quantities ranging from 1 to 8 mg of starting material were evaluated and analyzed using spectroscopic ellipsometry (Supporting Information‐Figure 3). A layer thickness of 63 ± 6 nm was observed for 2 mg of starting material. In further experiments, we tested surface‐bound films of **poly 3** for their ability to reinitiate the ROP, potentially opening the door for the synthesis of block co‐polymers. First, a **poly 3** film was prepared with a thickness of 54 nm on a PPX‐NH_2_‐coated substrate. The sample underwent a thorough washing procedure to remove adsorbed peptides [26, 27] (Supporting Information). The sample was then subjected to a subsequent cycle of vapor‐based ROP, which was reinitiated by the *N*‐termini of the original peptide chains. We observed a cumulative **poly 3** layer thickness of 198 nm as measured by spectroscopic ellipsometry (Supporting Information‐Figure 4a). Supporting Information‐Figure 4b depicts the IR spectrum of the initiator film **5** and the IR spectra of **poly 3** films formed during two consecutive SI‐VDP steps, showing a decrease in the intensities of the signals of the initiator film [57] **5** with increasing layer thickness of **poly 3**, indicating the successful ROP progression. Importantly, reinitiation was possible even after the sample had been stored in DMF for several days, pointing out the solvent stability of the presented multilayer polymer system.

Figure 1 summarizes the characterization of the synthesized NCA monomers as well as the corresponding surface‐bound polypeptides by IR, XPS, and SEM. The IR spectra of the NCAs **1** (gray), **2** (orange), and **3** (green) in Figure 1a confirm the presence of characteristic functional groups, such as the strong C≡C─H stretch vibration bands at around 3270 cm^−1^ and weak C≡C stretch vibration bands at around 2135 cm^−1^ (Supporting Information) [56, 61]. The ToF‐SIMS data of polypeptide films grown on different substrates were indistinguishable, suggesting that the tandem CVD process is indeed substrate‐independent. Characteristic bands of the PPX film **5** (i.e., the initiator layer) appear in the IR spectra of **poly 1–3**, although with lower intensity due to the presence of the polypeptide top layer. Furthermore, the amide I vibrations at around 1650 cm^−1^ appear as strong bands in the spectra of **poly 1** (gray), **poly 2** (orange), and **poly 3** (green). The IR spectra are primarily governed by the stretching vibrations of the C═O and C─N groups and depend on the secondary structure of the polypeptide backbone [62]. The amide bands A and B (generally between 3300  and 3070 cm^−1^), arising from the N─H stretch vibration, are also visible, but are superimposed by the signal of the C≡H stretching vibration in the spectrum of **poly 2** and **3**. Quantitative information about the elemental composition of the initiator layer 5 (Supporting Information‐Figure 1a) and the peptide films **poly 1–3** (Figure 1c) was obtained by XPS measurements. The chemical composition of the initiator layer matched the calculated values. The small amount of 3.3 at% oxygen likely resulted from radical quenching following the CVD polymerization. This observation is consistent with findings reported for other PPX coatings [63]. The XPS measurements of **poly 1–3** generally agree with calculated values, and minor deviations can be attributed to surface adsorption effects. A significant deviation from the theoretical value was observed in the measured values for aliphatic and aromatic carbons of **poly 3** (exp.: 48 at%, calc.: 60 at%). An explanation could be the partial thermal cleavage of the *O*‐propargyl residue, either during ROP or as a result of the XPS measurements. The topographies of **poly 1–3** were analyzed using SEM (Figure 1d), and variations are apparent, which appear to depend on the thickness of the resulting polypeptide films. In the case of **poly 3**, we observed the formation of island‐like structures starting from a poly(propargyltyrosine) thickness of approximately 44 nm. The islands appear broadly distributed and cover the entire substrate surface in the beginning. As the film growth progresses, the islands grow larger and larger and eventually crowd at a thickness of approximately 57 nm. At a critical limit of 65 nm, islands coalesce into a continuous film, forming ripple‐like structures [64, 65]. In contrast, a sample with a thicker peptide layer showed significant roughness and the absence of ordered structures (Supporting Information‐Figure 5, right column). Figure 1d displays SEM images of **poly 1** (left), **poly 2** (middle), and **poly 3** (right) with similar layer thicknesses. While **poly 3** displayed ordered topographic features, both **poly 1** and **poly 2** lacked similar structures. Their surfaces were dominated by irregular, island‐like topographic features, which could be attributed to the inability to generate film thicknesses of **poly 1** or **poly 2** as thick as those of **poly 3**. The effect might also appear at thicker layers.

Further analysis of the multilayer polymer system involved ToF‐SIMS depth profiling. This method enables a stepwise analysis of the polymer layers with increments of a few nanometers down to the gold substrate. Figure 2a illustrates the three‐dimensional distribution of specific ions throughout the polymer layers. The top row represents the depth profiles of the polypeptide layers **poly 1–3** (dark green) on PPX‐NH_2_ initiator films (blue) displaying the distribution of specific ions (shown are the fragments CN^−^, CNO^−^, and Au^−^). The CN^−^ fragments represent the PPX‐NH_2_ layer as well as the polypeptide films, whereas CNO^−^ is indicative of polypeptide structures. Au^−^ stems from the gold substrate used in this study. Although small amounts of oxygen were detected by XPS in the PPX‐NH_2_ layer, a distinct boundary was visible between the initiator layer (blue) and the overlying polypeptide layer (dark green, color codes for CN^−^ and CNO^−^ combined), indicating that the peptide layer was growing from the top of the initiator layer. Additionally, the depth profile of the **poly 3** sample (122 nm PPX‐NH_2_ + 57 nm polypeptide) showed a higher level of homogeneity compared to the other polypeptide samples, suggesting that NCA 3 films had significantly better quality than the other peptide films. It should be noted that the *z* direction is not to scale, due to the different erosion behavior of the individual polymer layers during ToF‐SIMS scanning, making a direct visual comparison of the thicknesses of the peptide layers of **poly 1**, **2**, and **3** impossible. Thicknesses of both the initiator layer and the polypeptide layers were measured by ellipsometry.

By employing the negative analyzer mode, we detected repeating units (RU^−^) as well as neighboring fragments ([RU + H]^−^ and [RU−H]^−^) that were specific for individual polypeptides. Figure 2b displays the intensities of the detected RU fragments for **poly 1** (on the left), **poly 2** (in the middle), and **poly 3** (on the right). As a reference, an untreated initiator sample was also included (as expected, their intensities were several orders of magnitude lower). For better visualization, we illustrated the distributions of the RUs for all three polypeptide films as 3D depth profiles (Supporting Information‐Figure 6), which followed the distributions of the CNO^−^ fragments represented in Figure 2a.

Based on these findings, we selected NCA **3** as the most promising candidate for exploring post‐polymerization modifications via micropatterning. Scheme 2 depicts the stepwise synthetic process from a sample pre‐coated with PPX to a micropatterned **poly 3** sample, featuring immobilized streptavidin Alexa Fluor 488 as the top layer. Biotin rapidly forms a complex with streptavidin [66, 67], which is stable over a broad range of biological conditions and, hence, is widely used in biomaterials science, e.g., in biosensors, for targeted drug delivery, or live cell imaging [68].

In this case, silicon substrates were coated with a 100 nm layer of PPX. Thereafter, structured PDMS stencils were applied on the resulting PPX substrates for the preparation of micropatterned initiator coatings (Scheme 2a, left) using vapor‐assisted micropatterning in replica structures (VAMPIR) [38, 69]. As a result, uniform and homogenous square patterns of PPX‐NH_2_ (300 × 300 µm) were created on a PPX background. White light interferometry (WLI) confirmed sharp and defined edges (Scheme 2a, right) and a step height of 115 nm between the top layer of the nonfunctional PPX background.

The micropatterned surfaces were then used for ROP of NCA **3** as described above (Scheme 2b, left). WLI analysis confirmed that the micropatterns remained intact after ROP of **3**. The difference between the top of the structural features and the PPX background was 248 nm, indicating a thickness of 132 nm for the **poly 3** film. After ROP of NCA **3,** the roughness within the squares increased from 1 ± 0.2 nm to 7 ± 3 nm, while the background remained unchanged (Scheme 2b right). Post‐polymerization modification involved 1,3‐dipolar cycloaddition between the polypeptide's alkyne groups and a biotinylated PEG‐azide, followed by the immobilization of streptavidin Alexa Fluor 488 (Scheme 2c, left) [69, 70, 71, 72]. After the reaction, the samples were thoroughly cleaned and analyzed using WLI and fluorescence microscopy (Scheme 2c, right and Supporting Information‐Figure 7). We observed a further increase in the height difference between the top of the squares and the background of approximately 33 nm. The 3D representation shows that even after several steps, the square patterns were well maintained with defined edges and smooth top layers. This highlights the robustness of the multilayer polymer system, an important feature for various biomedical applications, including protein, RNA arrays, or biosensors. To further investigate the selectivity of the multistep polymerization and binding reactions on structured surfaces, fluorescence microscopy was conducted. Supporting Information‐Figure 7 shows excellent contrast of the patterned **poly 3** films due to the spatially controlled immobilization of streptavidin Alexa Fluor 488. The low‐intensity background fluorescence has been attributed to nonspecific adsorption of streptavidin.

For a better understanding of the selectivity of the individual reaction steps, the micropatterned samples were also analyzed using ToF‐SIMS. Figure 3a shows the depth profiles of a square‐shaped vertical layer structure of a **poly 3** sample after the immobilization of the streptavidin conjugate as a 3D distribution of selected ions. The illustration on the left displays the theoretical vertical layer structure of a multilayered square area, with the biotin layer omitted for simplicity, followed by the overlayed visualization of the measured 3D distribution of Si^−^, CN^−^, CNO^−^, as well as the various sulfur‐containing fragments. Sulfur was used as a reporter for the biotin linker and streptavidin. As expected, the initiator layer is visible as the thickest layer based on a homogenous CN^−^ signal. The CNO^−^ signal (green) is primarily associated with the polypeptide layer as well as the streptavidin top layer. Sulfur‐containing ions were predominantly observed inside the squares (Figure 3a). The potential origin of sulfur‐containing fragments in the background could be nonspecific absorption of streptavidin or BSA, which is a component of the washing buffer. The characteristic signals of the repeating units of **poly 3** (RU^−^ = C_12_H_11_NO_2_^−^), along with its satellite fragments [RU−H]^−^ and [RU+H]^−^, were exclusively observed inside of the square patterns (yellow).

To obtain a clearer understanding of the selectivity of the reaction steps, we also present line scan plots of the ToF‐SIMS depth profile along with their corresponding intensity images in Figure 3b. The intensity plots of the CNO^−^ ions and the sulfur fragments clearly show that the intensities within the squares are several orders of magnitude higher than in the background, indicating high levels of selectivity in line with the results of the fluorescence microscopy.

## 3 Conclusions

In summary, we have developed and validated a tandem CVD platform for the solvent‐free synthesis of functional, surface‐bound peptide architectures. The NCAs were selected for their orthogonal functionality, allowing direct post‐polymerization modifications without the need for protecting groups. *O*‐propargyl‐(*S*)‐tyrosine‐NCA was used to demonstrate further chemical modifications. This approach resulted in micropatterned poly(propargyltyrosine) surfaces, which supported the straightforward immobilization of Alexa Fluor 488‐functionalized streptavidin. This highly versatile tandem CVD platform complements existing methods for synthesizing surface‐bound polypeptide coatings, potentially paving the way for complex peptide‐based biomaterial architectures. Future experiments are required to upscale the tandem CVD process and to apply it to medically more relevant substrates. Further expansion of the structural diversity of NCAs that can be processed using this approach will enhance its versatility and will be required to establish a powerful surface modification platform.

## Conflicts of Interest

The authors declare no conflicts of interest.

## Supporting information




**Supplementary File 1**: chem70697‐sup‐0001‐SuppMat.docx.
