# Supplementary material for: A Tandem Chemical Vapor Deposition Platform for the Solvent‐Free Synthesis of Polypeptide Architectures
Source: Chemistry. 2026 Jan 19;32(12):e03611. doi: 10.1002/chem.202503611 (PMC13037357; doi:10.1002/chem.202503611)
Supplement: Supplementary file 1 — chem70697‐sup‐0001‐SuppMat.docx. [file CHEM-32-e03611-s001.docx]

**Supporting Information**

**Experimental section**

**Materials**

The amino acids *O*-Propargyl-(*S*)-tyrosine and propargyl-(*S*)-glycine were purchased from Iris Biotech (Marktredwiz, Germany). Streptavidin Alexa Fluor™ 488 was purchased from Fisher Scientific (Schwerte, Germany). Triphosgene, 2-methyloxiran, Biotin-dPEG®7-azide, THF, CuSO_4_, ascorbic acid, bovine serum albumin (BSA), Dulbecco´s phosphate buffered saline (DPBS), and hexane were purchased from Sigma-Aldrich Chemie (Taufkirchen, Germany). [2.2]Paracyclophane was purchased from TCI (Eschborn, Germany). All chemicals and materials were used as received unless otherwise stated. Gold wafers with 100 nm thickness on [100] silicon wafers precoated with titanium were purchased from Georg Albert PVD-Beschichtung (Silz, Germany). Silicon wafers were obtained from Si-Mat Silicon Materials (Kaufering, Germany). The wafers were cleaned before use by washing with acetone and ethanol and subsequent drying under an argon stream. Electronic components in the experimental setup for the synthesis of polypeptide films were purchased from RS Components Deutschland (Frankfurt a. M., Germany) and Innovative Sensor Technology IST AG (Ebnat-Kappel, Switzerland). Components for the custom-built chemical vapor deposition device are from Kurt J. Lesker Company (Dresden, Germany).

**Characterization**

**NMR** (nuclear magnetic resonance) spectra were recorded on a Bruker Ascend III 400 MHz spectrometer (Massachusetts USA) at a frequency of *ν* = 400 MHz (^1^H) or *ν* = 101 MHz (^13^C). All coupling constants are absolute values and *J* values are expressed in Hertz (Hz). The description of the signals includes: s = singlet, bs = broad singlet, d = doublet, t = triplet, q = quartet, dd = doublet of doublet, ddd = double doublet of doublet, m = multiplet. References for ^1^H NMR and ^13^C NMR spectra were the residual solvent peaks of chloroform (^1^H: δ = 7.26 ppm, ^13^C: δ = 77.16 ppm) and acetone (^1^H: δ = 2.05 ppm, ^13^C: δ = 29.84 ppm), which were purchased from Sigma-Aldrich Chemie (Taufkirchen, Germany). The spectra were analyzed according to first order. The assignment of signals in ^13^C spectra of *O*-propargyl-(*S*)-tyrosine-NCA (**3**) was performed using DEPT90 or DEPT135 spectra (DEPT = distortionless enhancement by polarization transfer). The signal assignments are as follows: positive DEPT signal (+): primary or tertiary carbon atoms, negative DEPT signal (-): secondary carbon atoms. Quaternary carbon atoms (C_quart_) do not display a DEPT signal.

**ToF-SIMS** (time-of-flight secondary ion mass spectrometry) was performed on a TOF.SIMS5 instrument (ION-TOF GmbH, Münster, Germany). This spectrometer is equipped with a Bi liquid cluster primary ion source and a reflection type time-of-flight analyzer. UHV base pressure was < 5 × 10^-8^ mbar. For high mass resolution the Bi source was operated in the “high current bunched” mode providing 1.2 - 1.38 ns Bi_3_^+^ primary ion pulses at 25 keV energy, a lateral resolution of approx. 4 μm, and a target current of 0.35 pA at 100 µs cycle time. The primary ion beam was scanned across 300 × 300 µm^2^ field of view on the sample, and 128 × 128 data points were recorded. Spectra were calibrated on C^-^, CH^-^, O^-^, OH^-^, C_2_^-^, C_3_^-^ and C_3_H_2_^-^ signals. Based on these datasets, the chemical assignments for characteristic fragments were determined.

For depth profiling a dual beam analysis was performed in non-interlaced mode. The sputter gun operated with Ar_1300_^+^ ions, 5 keV, scanned over a concentric field of 750 x 750 µm^2^, target current 2.3 - 2.7 nA, was applied to erode the sample. The primary ion beam was scanned across a 300 × 300 µm^2^ field of view centered in the crater, and 128 × 128 data points were recorded with a target current of 0.34 - 0.38 pA at 100 µs cycle time. The shift of the calibration due to unexpected charging effects during the depth profiling was corrected using Advanced ToF Correction (Depth) of the onboard software. Due to the high sensitivity of the technique and for better visualization of the different structural sections, an intensity cutoff of 40 % was used for fragments with high intensity (CN^-^, CNO^-^) in the three-dimensional view. For fragments with low intensity, a value of 5 – 15 % was used.

**XPS** (X-ray photoelectron spectroscopy) measurements were performed under ultra-high vacuum conditions with a base pressure of 1 × 10^–9^ mbar. Core-level spectra were recorded under normal emission with a Scienta R4000 hemispherical electron analyzer using Mg-K_α_ radiation (1253.7 eV). First, for every sample the survey XP spectrum was measured and no unexpected contaminations were observed in these spectra. Then, the narrow scan of N1s, C1s, O1s, and Au4f_7/2_ XP spectra were recorded. For a precise determination of the different line positions and necessary corrections, the CasaXPS Version 2.3.5PR1.0 was used.^[1]^ All spectra were fitted with 50/50 % of Lorentz-Gaussian contribution using Shirley background for all peaks. The binding energy (B. E.) was calibrated by setting the C1s peak to 285.0 eV. Peaks were calibrated according to the literature.^[2]^

**IRRAS** (infrared reflection–absorption spectroscopy) of polymer films on gold samples was performed with a Bruker VERTEX 80 FTIR (Bruker Optik GmbH, Ettlingen, Germany). The spectra were scanned with a resolution of 2 cm^-1^ over the range of 800 – 4000 cm^-1^. The device was equipped with a horizontal reflection unit for measurements in grazing incidence reflection mode with an incident angle of 80° to the surface normal background correction was performed by the onboard software Bruker OPUS.

**SEM** (scanning electron microscopy) images were acquired using a Philips XL 30 field emission gun environmental scanning electron microscope (FEG-ESEM) (Philips Electron Optics, Eindhoven, The Netherlands) with a software and hardware update of point electronic (point electronic GmbH, Halle, Germany) operated at an accelerating voltage of 5 kV in the high-vacuum mode. The samples were mounted on an aluminum stud using adhesive carbon tape and were sputter-coated with approximately 3 nm platinum using a BAL-TEC MED 020 sputter coater connected to a BALTEC MCS 010 and a BAL-TEC QSG 060 (BAL-TEC AG, Pfäffikon, Switzerland). For better visualization of the topographical structure, Secondary Electron (SE) and Back Scattered Electron (BSE) images were mixed in topo/compo mode with the channel mixer of the onboard software. SEM measurements were also performed on a VEGA3 device (TESCAN GmbH, Dortmund, Germany) using the VEGA3 control software 4.2.24.0 build 1262 (WD ~7 mm, SEM HV 10 kV, beam intensity 5).

**Epi-fluorescence microscopy** was performed on an upright microscope (Axio Imager.M2, Zeiss, Germany) equipped with a light-emitting diode (LED) light source (Colibri 7, Zeiss, Germany). The samples were measured using an LED module with an emission wavelength of 475 nm (intensity 100 %, 450 nm – 488 nm) and a Plan-Neofluar 10x/0.30 Ph1 or EC Plan-Neofluar 2.5x/0.085 M27 objective, with the 110 HE (DAPI/GFP/Cy3.5/cy7) reflector/filter cube.

**ATR‑FTIR** (attenuated total reflectance Fourier-transform infrared-spectroscopy) measurements of polymer films on gold samples as well as of solid precursors were performed on a Tensor 27 spectrometer (Bruker Optik, Ettlingen, Germany) equipped with a Bruker Optics Platinum ATR accessory (diamond crystal, 1 mm^2^ area with single reflection). The absorption bands were given in wave numbers *ν* in cm^-1^ and the forms and intensities of the bands were characterized as follows: s = strong ≥ 70 % Absorbance (A), m = medium 70 – 40 % A, w = weak ≤ 40 % A.

**LC-MS** (liquid chromatography–mass spectrometry): The separation of the target compound was performed by using an ExionLC AD system (Shimadzu) equipped with a Luna Omega C18, 1.6 μm, 2.1 x 100 mm column (Phenomenex) which was coupled to an X500R mass spectrometer (AB Sciex). A total runtime of 12 min was applied, starting at 80 % of eluent B (water) and 20 % of eluent A (acetonitrile) for 1 min, followed by a linear gradient to 80 % of eluent A over 5 min, which was held for 4 min and switched back to the starting conditions to equilibrate the column for the next run. The flow rate was constant at 300 μL/min and all analyses were carried out using a sample volume of 5 μL. Mass spectrometric analyses were done by using electrospray ionization (ESI) operating in the negative ion mode. The ion source was heated up to 500 °C and an ion spray voltage of -4500 V was applied. MS-experiments were carried out at a collision energy (CE) of ‑10 V and a declustering potential (DP) of ‑35 V. The acquisition range was 60 to 400 m/z. In all experiments nitrogen gas 5.0 was used as nebulizer, curtain and collision gas. The resulting chromatograms were evaluated with SCIEX OS 2.1.0 instrument software.

**White Light Interference (WLI)** Measurements and Confocal Laser Microscopy images were taken using a Keyence VK-X3000 3D Laser Scanning Microscope (Keyence Deutschland GmbH, Neu-Isenburg, Germany). The images were background corrected applying a linear tilt correction in xy-direction using the instrument software.

**Spectroscopic ellipsometry measurements** were conducted using an M2000 instrument from J.A. Woollam Co. Inc., based in Lincoln, NE, USA. The measurements were carried out at an angle of incidence of 70° within the spectral range of 370 nm – 1000 nm under environmental conditions. An optical box model was used, where the optical properties of the transparent polymers were fitted by a Cauchy function, utilizing the instrument's software CompleteEase (V5.19). The optical characteristics of the gold-coated silicon substrate were determined through a reference measurement of the uncoated substrate performed prior to the synthesis.

**Precursor synthesis**

The precursor for the preparation of the initiator coating, 4,16-diamino[2.2]paracyclophane (**4**), was synthesized from [2.2]paracyclophane in a three-step reaction according to a literature-known procedure.^[3]^ (*S*)-Phenylalanine-NCA (**1**), propargyl-(*S*)-glycine-NCA (**2**) and *O*-propargyl-(*S*)-tyrosine-NCA (**3**) were synthesized using a general synthetic method published by Tian *et* *al*.^[4]^ The method was adapted for the amino acids (*S*)-phenylalanine, propargyl-(*S*)-glycine, *O*-propargyl-(*S*)-tyrosine, with slight modifications in the workup. The crude NCA products were dissolved in 5 mL THF and precipitated in cold hexane and then recrystallized from a mixture of cold hexane and THF. Afterwards, the purified NCAs were stored under argon at -20 °C and utilized as soon as possible.

(*S*)-Phenylalanine-NCA (**1**) was obtained in a yield of 80 %. – ^1^H-NMR (400 MHz, CDCl_3_): δ = 7.38 – 7.30 (m, 3H, H_Ar_), 7.20 – 7.18 (m, 2H, H_Ar_), 6.04 (bs, 1H, NH), 4.53 (ddd, *J* = 8.4, 4.1, 0.9 Hz, 1H, CH), 3.29 (dd, *J* = 14.1, 4.1 Hz, 1H, CH_2_), 3.00 (dd, *J* = 14.1, 8.4 Hz, 1H, CH_2_) ppm. ^13^C-NMR (100 MHz, CDCl_3_): δ = 168.7 (C=O), 151.9 (C=O), 134.1 (C_Ar_), 129.4 (2 x C_Ar_), 129.3 (2 x C_Ar_), 128.2 (C_Ar_), 58.9 (O-[C=O]-*C*H), 38.0 (CH_2_) ppm. IR (ATR): *ν* = 3280 (m), 1848 (m), 1755 (s) cm^‑1^. The data agree with the literature.^[4-5]^

Propargyl-(*S*)-glycine-NCA (**2**) was obtained in a yield of 70 %. – ^1^H-NMR (400 MHz, acetone-d^6^): δ = 8.04 (s, 1H, NH), 4.75 (t, *J* = 4.5 Hz, 1H, CH), 2.84 (dd, *J* = 4.5, 2.4 Hz, 2H, CH_2_≡C), 2.60 (t, *J* = 2.7 Hz, 1H, C≡CH) ppm. ^13^C-NMR (100 MHz, acetone-d^6^): δ = 170.6 (-O-[C=O]CH), 152.7 (-O-[C=O]NH), 78.2 (-C≡), 73.6 (≡CH), 57.4 (CH), 22.3 (CH_2_) ppm. IR (ATR): *ν* = 3267 (m, C≡H stretch), 2141 (w, C≡C stretch), 1809 (m), 1755 (s) cm^‑1^. MS (ESI-ToF) [*m/z]*: exact mass 139.0269 [M], found 138.0158 [M – H]^–^. The data agree with the literature.^[6-7]^

*O*-Propargyl-(*S*)-tyrosine-NCA (**3**) was obtained in a yield of 75 %. – ^1^H-NMR (400 MHz, acetone-d^6^): δ = 7.94 (s, 1H, NH), 7.18 (d, *J* = 8.7 Hz, 2H, H_Ar_), 6.92 (d, *J* = 8.7 Hz, 2H, H_Ar_), 4.79 (ddd, *J* = 5.9, 4.8, 1.0 Hz, 1H, CH), 4.74 (d, *J* = 2.4 Hz, 2H, OCH_2_), 3.18 – 3.04 (m, 2H, CH_2_), 3.03 (t, *J* = 2.4 Hz, 1H, C≡CH) ppm. ^13^C-NMR (100 MHz, acetone-d^6^): δ = 171.21 (C_quart_, C-O-[*C*=O]CH), 158.0 (C_quart_,C_Ar_-O), 152.4 (C_quart_,-O-[*C*=O]NH), 131.7 (+, 2 x C_Ar_), 128.5 (C_quart_, C_Ar_), 115.7 (+, 2 x C_Ar_), 79.8 (+, -C≡), 76.9 (+, ≡CH), 59.7 (+, CH), 56.2 (–, O-CH_2_), 36.9 (–, C_Ar_-*C*H2) ppm. IR (ATR): 3282 (m, C≡H stretch), 2131 (w, C≡C stretch), 1857 (m), 1779 (s) cm^‑1^. MS (ESI-ToF) [*m/z]*: exact mass 245.0688 [M], found 244.0618 [M – H]^–^.

**Sample preparation**

**Initiator coatings** were prepared via CVD polymerization of 4,16-diamino[2.2]paracyclophane (**4**) using a custom-made experimental setup established by our group:^[8-9]^: 50 mg precursor was sublimed under a reduced pressure of 0.125 mbar at approximately 110 °C. The sublimed precursor was then transported into the furnace using Argon as carrier gas at a constant flow rate of 20.0 sccm. The precursor was converted into the corresponding quinodimethane fragments at a pyrolysis temperature of 660 °C. Deposition and polymerization of the fragments were carried out by further transport into the deposition chamber on rotating (30 rpm) pre-cut (12 x 12 mm) gold or silicon substrates, which were cooled to 15 °C. A constant deposition rate of 0.2 – 0.6 A s^−1^ was maintained throughout the deposition process, monitored by a deposition controller (XTC/3, Inficon GmbH, Cologne, Germany). The wall of the deposition chamber was heated to a constant temperature of 73 °C to prevent residual deposition on the chamber walls. The resulting poly(amino-*p*-xylyen) films (PPX-NH_2_, **5**) were cleaned with acetone and ethanol and dried under argon flow. Micropatterned surfaces were obtained using pre-structured PDMS micro stencils (square size: 300 x 300 µm, inter-square distance: 300 µm) on substrates during deposition, following the published VAMPIR (vapor-assisted micropatterning in replica structures) technique.^[10]^ PDMS stencils were prepared using a standardized and literature-known procedure.^[10]^ In order to reach sufficient deposition through the holes of the stencils, about twice the amount of precursor was needed to reach a similar layer thickness as in the unmasked deposition, due to the shadowing effect of the mask.

**Polypeptide films** were prepared through SI-VDP of (*S*)-Phenylalanine-NCA (**1**), propargyl-(*S*)-glycine (**2**) or *O*-Propargyl-(*S*)-tyrosine (**3**), using a custom-made cylindric glass reaction chamber with a total volume of approximately 80 mL (SI-Figure 2). A modified ground glass joint with a rubber septum and a thin glass tube of approximately 5 mm inner diameter and 70 mm length, mounted vertically, served as a holder for both the micro heating plate and the sample. The micro heating plate (10 x 10 mm, 14.0 Ω) and its thermocouple (PT100 class F0.1) were mounted at the bottom of the glass tube, held by cables leading out of the reaction chamber through the glass tube and the rubber septum, connected to an external temperature controller. Precoated initiator samples (12 x 12 mm) were attached to the micro heating plate with the initiator side facing downwards using double-sided copper tape. To prevent unspecific deposition on the sample holder, glass tube, or cables, a layer of Teflon tape was applied prior to each run serving as a barrier. The corresponding amount of powdered NCA (NCA **1**: 15 – 30 mg, NCA **2**: 25 – 45 mg, NCA **3**: 1 – 8 mg) was placed at the bottom of the reaction chamber, which was then sealed with the described ground joint using a Teflon ring to ensure proper sealing. With this experimental setup, the sample was positioned 30 mm above the NCA source. The reaction chamber was then connected to the Schlenk-line via a hose connection and was evacuated and backfilled with argon three times. After the final evacuation, the pressure in the reaction chamber was 1 x 10^‑4^ mbar, and the heating plate was set to the desired deposition temperature (NCA **1**: 60 °C, NCA **2**: 60 °C, NCA **3**: 80 °C). To start the reaction, the flask was immersed 5 mm into the oil bath preheated to the desired evaporation temperature (NCA **1**: 95 °C, NCA **2**: 110 °C, NCA **3**: 142 °C). The reaction chamber was continuously evacuated during the polymerization to maintain constant pressure and to remove the CO_2_ formed during the reaction. After the reaction (NCA **1**: 25 min, NCA **2**: 10 min, NCA **3**: 10 min), the sample was carefully removed from the heating plate and thoroughly washed with acetone and ethanol. The samples of **poly 1 – 3** were then placed in chloroform containing 10 vol % dichloroacetic acid (DCA) for 1 h, washed with chloroform and placed in DMF for several days, to remove physisorbed oligopeptides.^[11-12]^ The samples were finally washed with DMF, ethanol, and dried in an argon stream. The reaction vessel used in the presented work differed considerably in volume and construction as compared to the previous reports, pointing out the dependency of the reaction conditions on the nature of the experimental setup.^[13]^

**The fluorophore** was attached to micro-structured alkyne-functional peptide films following a two-step procedure with slight modifications.^[14]^ Biotin-dPEG®7-azide was first bonded to the alkyne groups via copper-catalyzed azide-alkyne cycloaddition (CuAAC) followed by the attachment of Streptavidin-Alexa Fluor™488 conjugate (Molecular Probes™) via specific biotin/streptavidin interaction. For that purpose, Biotin-dPEG®7-azide (10 mg/mL, 1.00 eq) was dissolved in demineralized water. Next, 0.20 eq CuSO_4_ **^.^** 5 H_2_O, 0.20 eq THPTA, and 0.40 eq ascorbic acid were added resulting in a light green, clear solution. Finally, the reaction mixture was transferred into 10 mL snap-top glass vials, containing the alkyne-functional polypeptide surfaces. After careful shaking for 18 h at room temperature, the samples were removed and thoroughly washed with demineralized water and ethanol and dried in an argon stream. In the final step, the samples were incubated in DPBS containing 0.1 % (w/v) BSA and 0.02 % (v/v) Tween 20, along with the streptavidin conjugate at a concentration of 10 µg/mL. After another 18 hours of gentle shaking at room temperature, the samples were removed and rinsed with DPBS containing 0.1 % (w/v) BSA and 0.02 % (v/v) Tween 20.

**Figures**


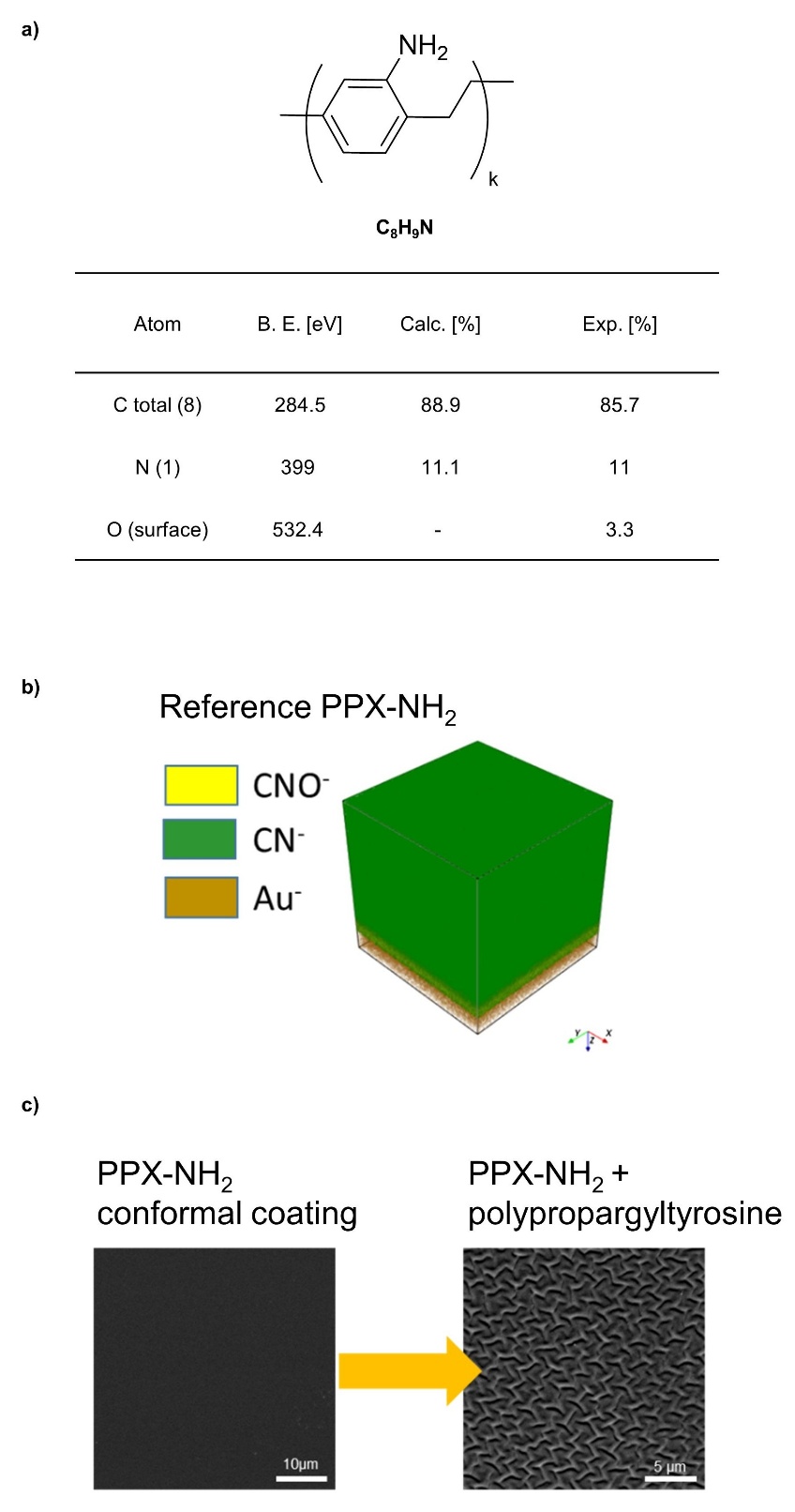


**SI-Figure 1.** Chemical composition of the PPX-NH2 initiator layer, determined by XPS and TOF-SIMS.

**
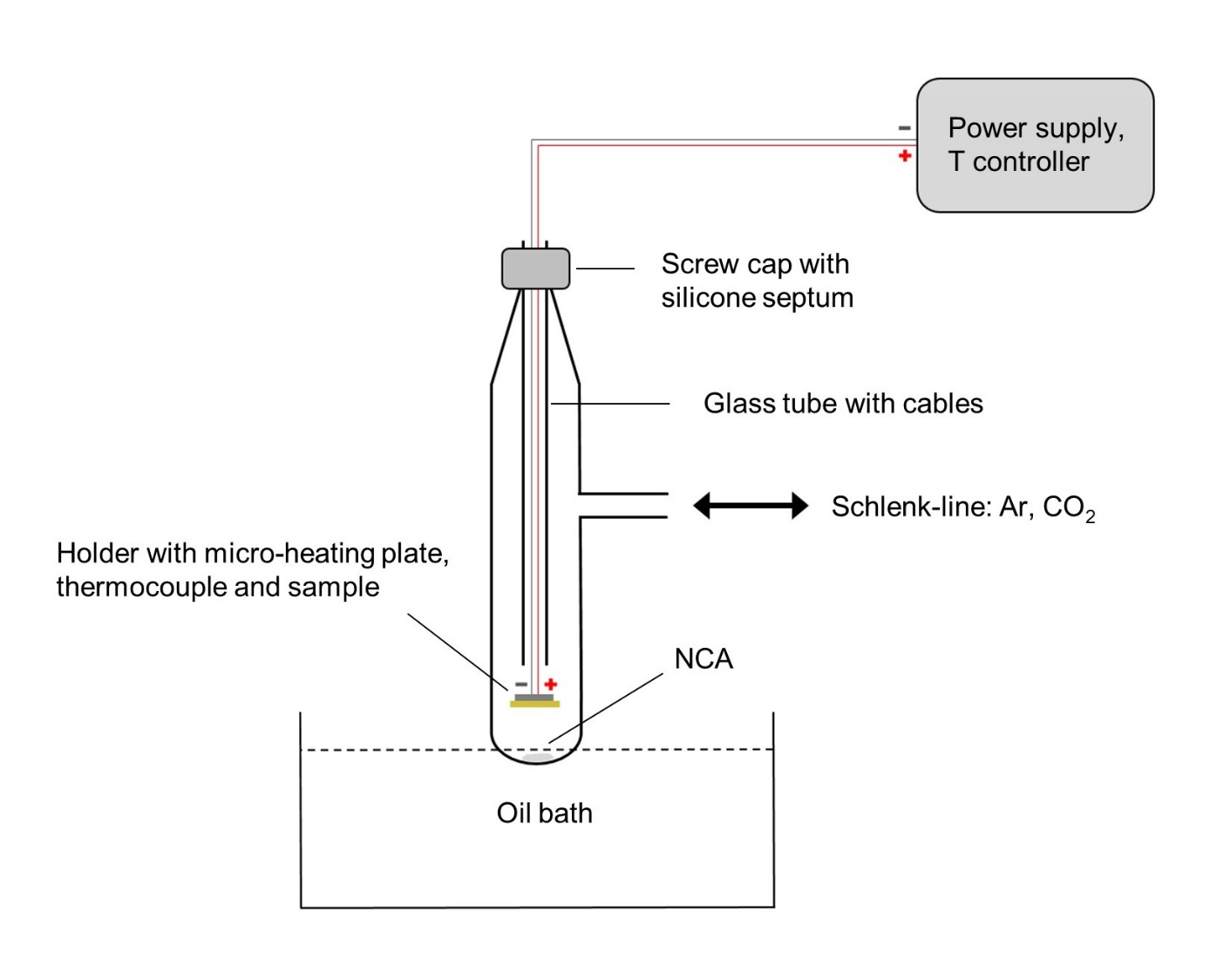
**

**SI-Figure 2.** Experimental setup for the surface-initiated vapor deposition polymerization of functionalized NCAs: The ring-opening polymerization was carried out in a custom-made reaction chamber using an oil bath as the heat source for the evaporation of the NCAs. The vessel was equipped with a capillary glass tube connected to a rubber septum to operate the micro heater inside the evacuated reaction chamber during the reaction. Using double-sided copper glue tape, the initiator samples were mounted to the micro-heating plate facing downwards.

**
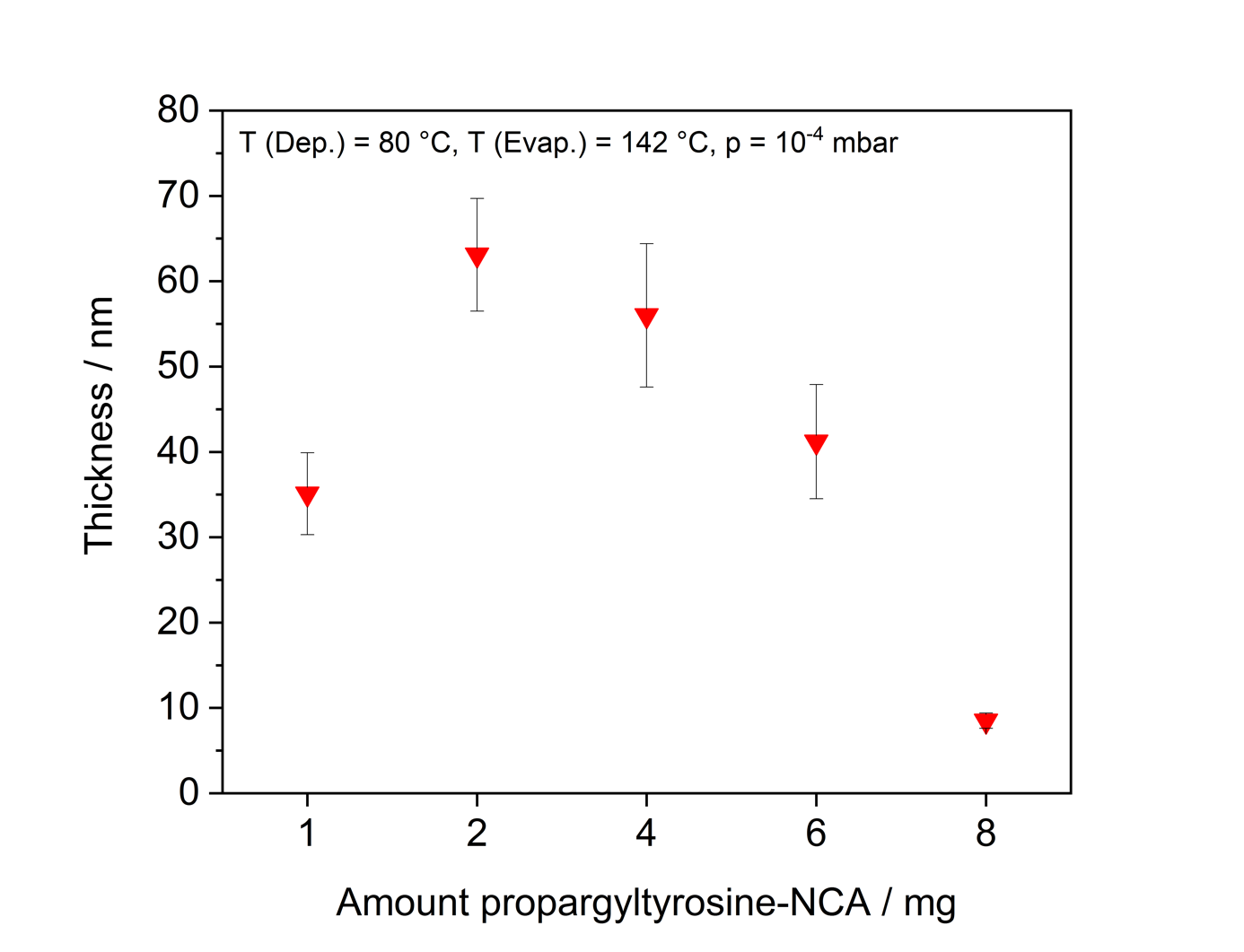
**

SI-Figure 3. Film thickness of surface-bound poly 3 films measured by ellipsometry versus different amounts of starting material after a reaction time of 10 minutes. Each measuring point represents an average of four runs

**
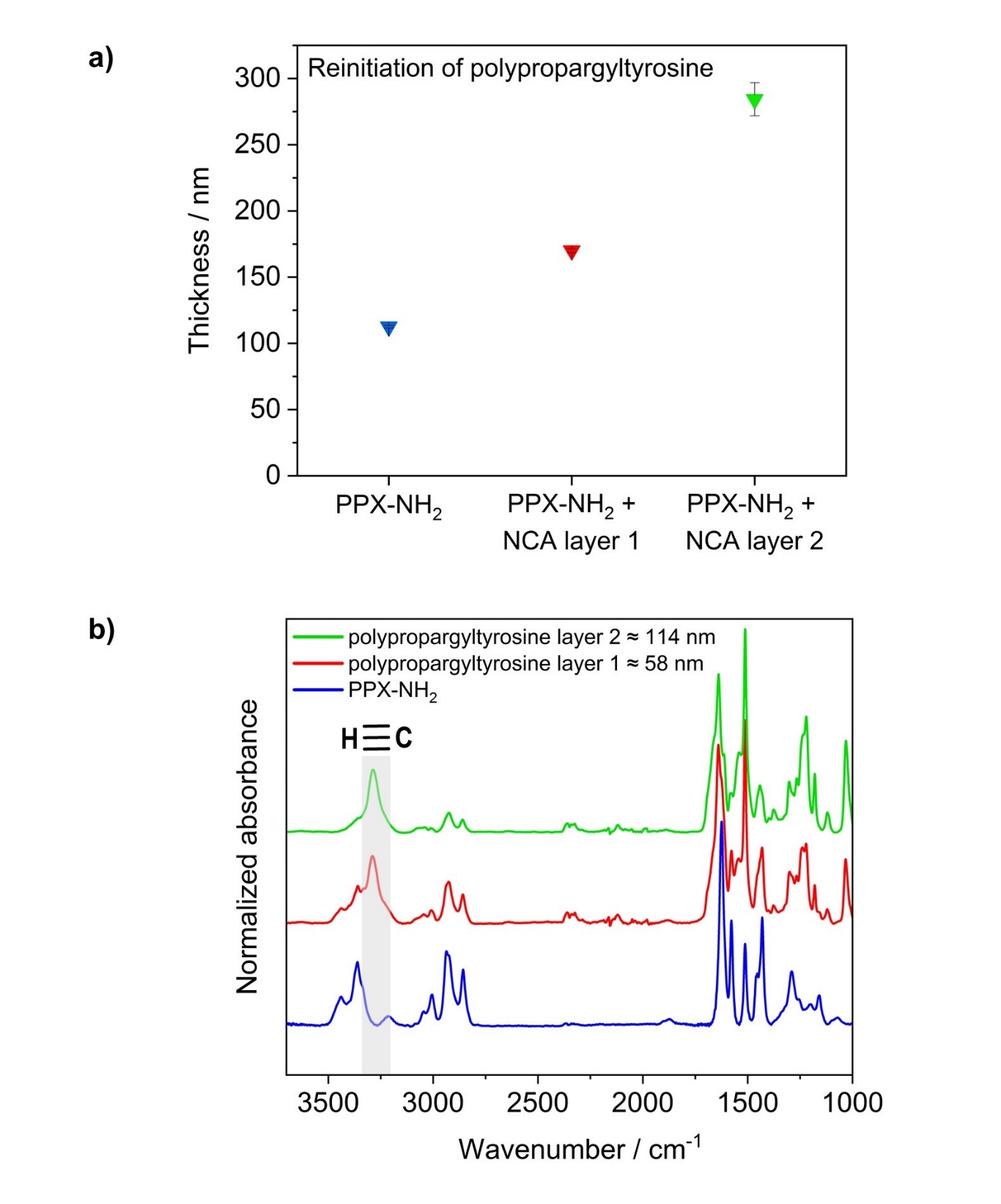
**

**SI-Figure 4.** Reinitiation of **poly 3**: a) film thickness of different polymer layers measured by ellipsometry. The first layer of **poly 3** was polymerized on a pre-coated PPX-NH_2_ substrate with a thickness of 112 nm. The terminal amine groups of the resulting polymer film served as initiators for the attachment of a second **poly 3** layer resulting in a total thickness of approximately 284 nm. b) IR-spectra of the corresponding polymer layers showing an increase in intensity of the signal assigned to the C≡H stretch vibrations of **poly 3**.

**
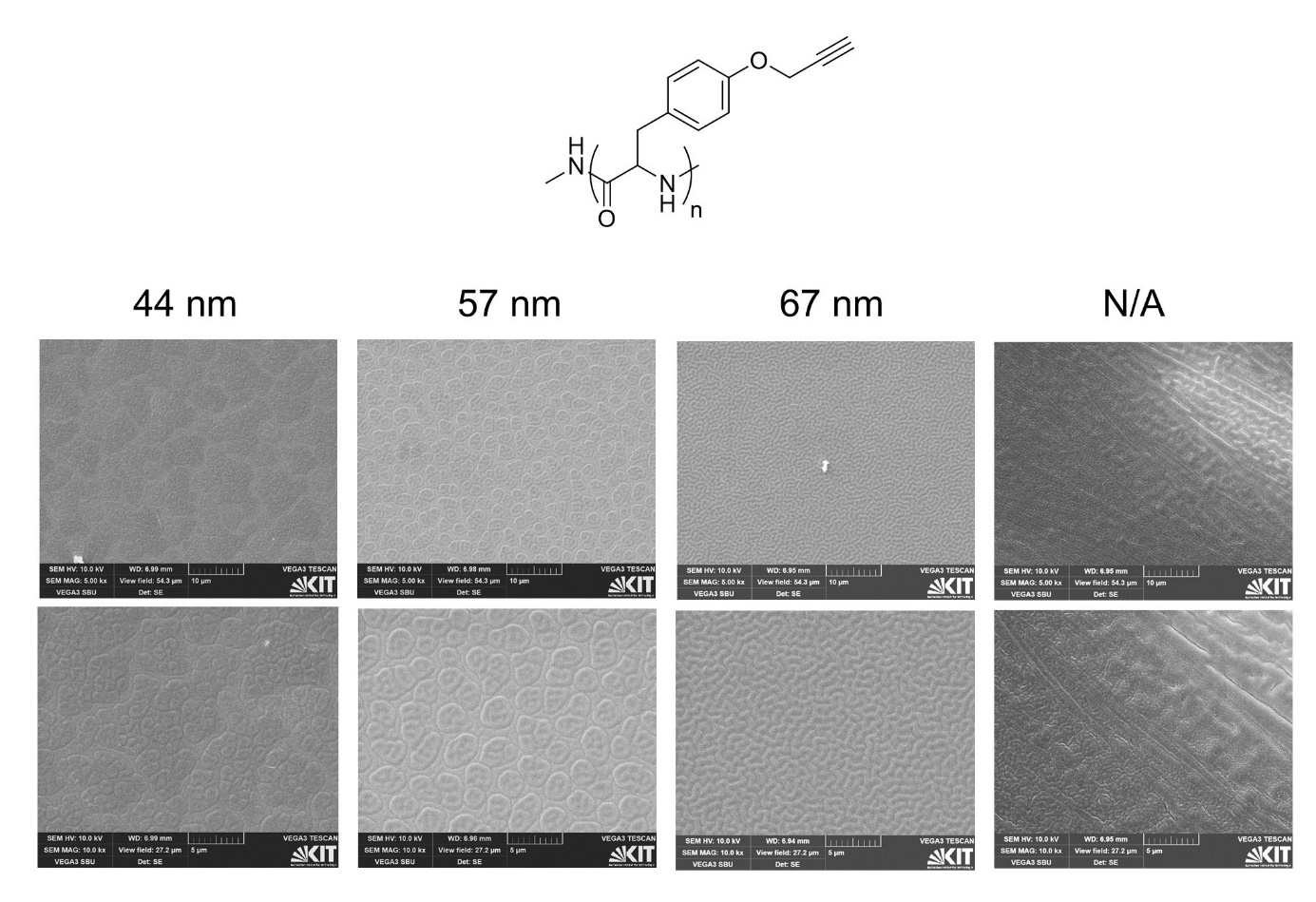
**

SI-Figure 5. SEM images (SE and BSE combined) of poly 3 films at different magnifications arranged vertically displaying the dependency between film thickness and surface morphology. The film thickness values were measured by ellipsometry and could not be determined for the sample depicted in the right column due to the polypeptide layer's lack of transparency.


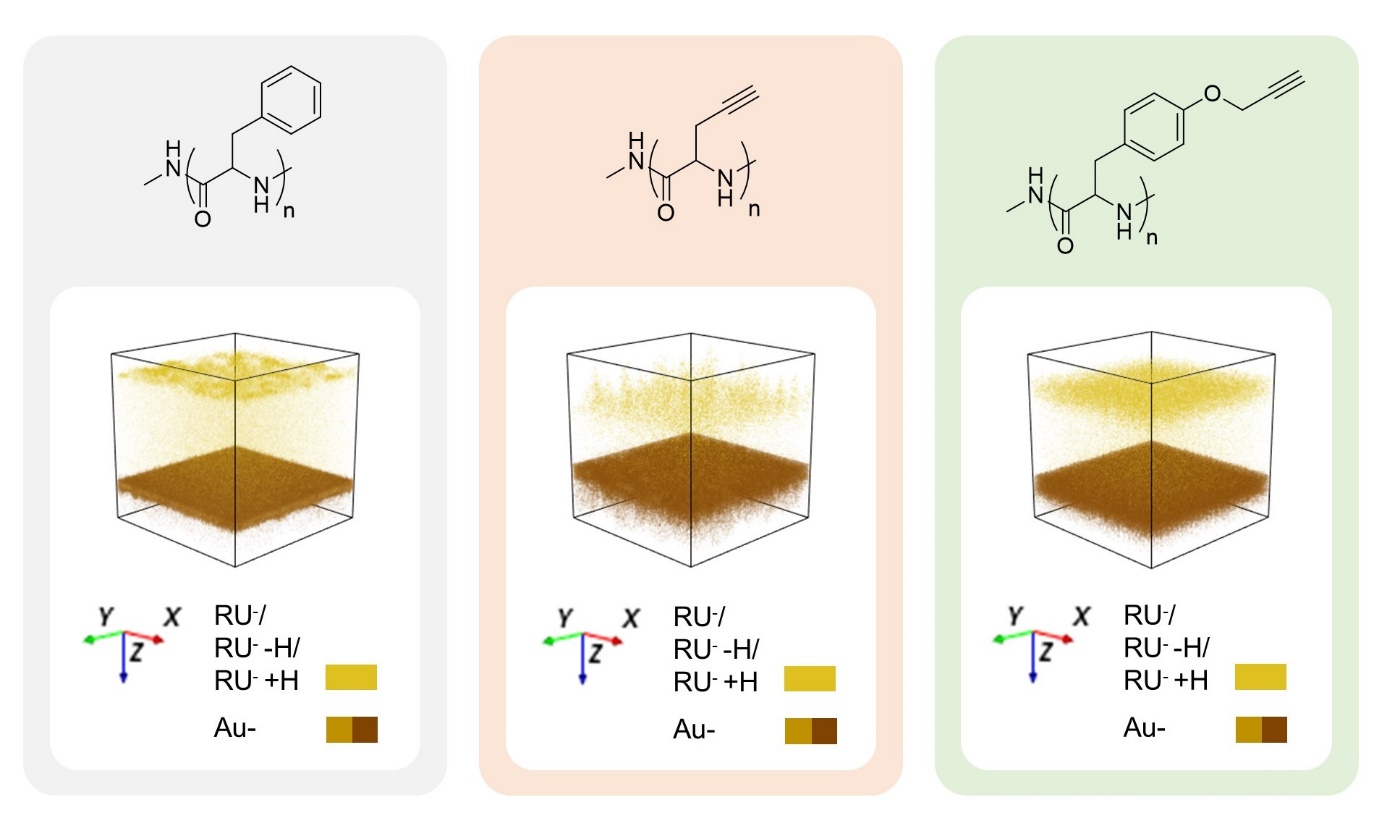


**SI-Figure 6.** Advanced analysis of the multi-component system using ToF-SIMS: depth profiles displaying the three-dimensional main distribution of the detected repeating unit (RU^-^) and its neighboring fragments (RU^-^+ H, RU^-^-H) of **poly 1** (left), **poly 2** (middle) and **poly 3** (right), x and y 300 µm, z not to scale.


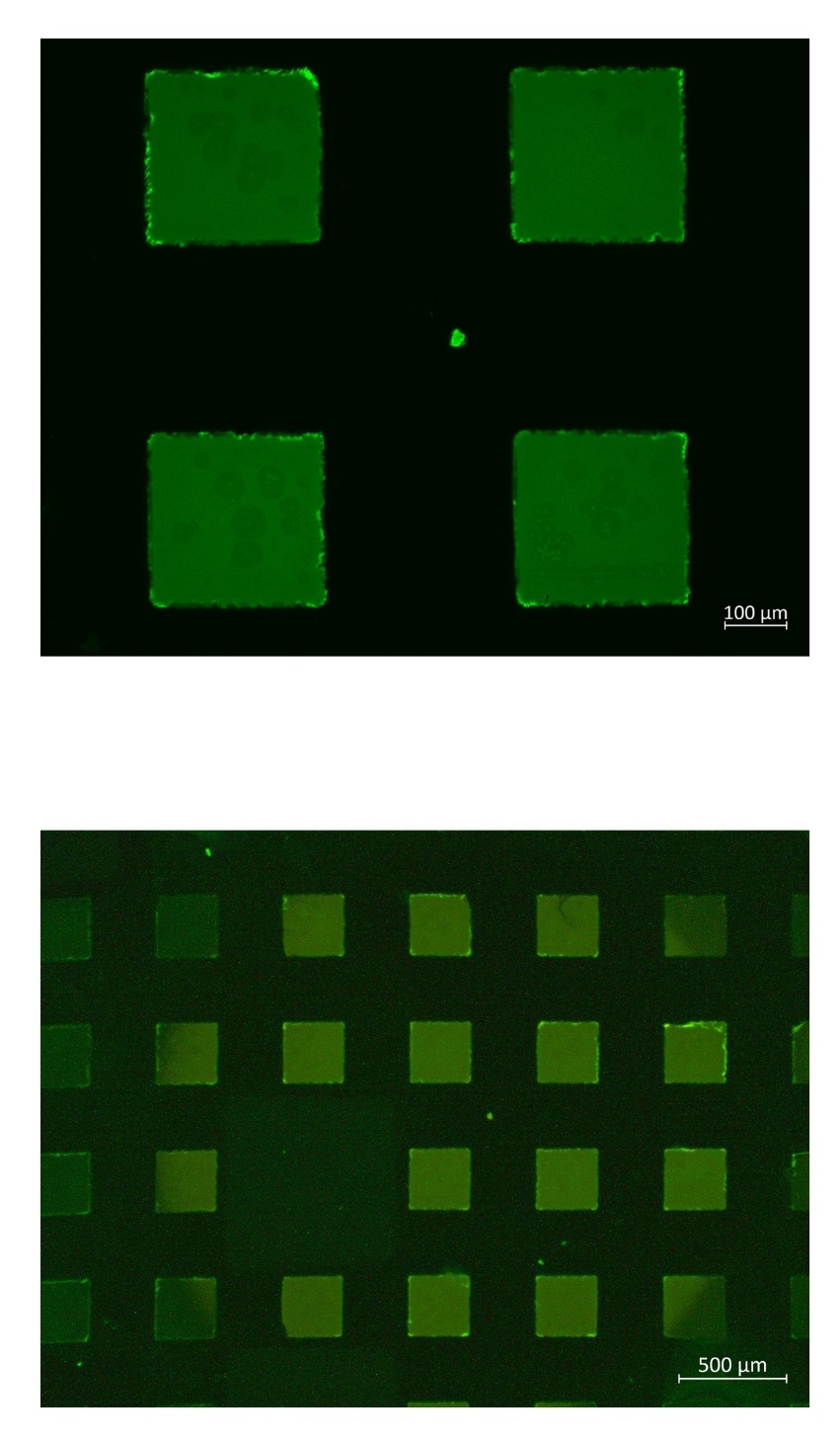


SI-Figure 7. Fluorescence micrographs in different magnifications of a micro-structured poly 3 film prepared via the VAMPIR technique after the immobilization of streptavidin Alexa-Fluor™ 488. The area of the missing square in the bottom image was used for ToF-SIMS analysis.

[1] Fairley, N.; Fernandez, V.; Richard‐Plouet, M.; Guillot-Deudon, C.; Walton, J.; Smith, E.; Flahaut, D.; Greiner, M.; Biesinger, M.; Tougaard, S.; Morgan, D.; Baltrusaitis, J., Systematic and collaborative approach to problem solving using X-ray photoelectron spectroscopy. *Applied Surface Science Advances* **2021,** *5*, 100112.

[2] Moulder, J. F.; Stickle, W. F.; Sobol, W. M.; Bomben, K. D. In *Handbook of X-Ray Photoelectron Spectroscopy*, 1992.

[3] Lahann, J.; Höcker, H.; Langer, R., Synthesis of Amino[2.2]paracyclophanes-Beneficial Monomers for Bioactive Coating of Medical Implant Materials. *Angew Chem Int Ed Engl* **2001,** *40* (16), 2947.

[4] Tian, Z.-Y.; Zhang, Z.; Wang, S.; Lu, H., A moisture-tolerant route to unprotected α/β-amino acid N-carboxyanhydrides and facile synthesis of hyperbranched polypeptides. *Nature Communications* **2021,** *12* (1), 5810.

[5] Zovko Končić, M.; Kalčić, I.; Zorc, B.; Butula, I., IR spectroscopy study of the amidation reaction of N-(1-azolecarbonyl) amino acids on L-phenylalanine derivative models. *Croatica Chemica Acta* **2003,** *76*, 229-233.

[6] Huang, J.; Bonduelle, C.; Thévenot, J.; Lecommandoux, S.; Heise, A., Biologically Active Polymersomes from Amphiphilic Glycopeptides. *Journal of the American Chemical Society* **2012,** *134* (1), 119-122.

[7] Coates, J., Interpretation of Infrared Spectra, A Practical Approach. In *Encyclopedia of Analytical Chemistry*.

[8] Lahann, J., REACTIVE POLYMER COATINGS FOR BIOMIMETIC SURFACE ENGINEERING. *Chemical Engineering Communications* **2006,** *193* (11), 1457-1468.

[9] Chen, H.-Y.; Lahann, J., Designable Biointerfaces Using Vapor-Based Reactive Polymers. *Langmuir* **2011,** *27* (1), 34-48.

[10] Chen, H.-Y.; Lahann, J., Vapor-Assisted Micropatterning in Replica Structures: A Solventless Approach towards Topologically and Chemically Designable Surfaces. *Advanced Materials* **2007,** *19* (22), 3801-3808.

[11] Wang, Y.; Chang, Y.-C., Grafting of Homo- and Block Co-polypeptides on Solid Substrates by an Improved Surface-Initiated Vapor Deposition Polymerization. *Langmuir* **2002,** *18* (25), 9859-9866.

[12] Lee, N. H.; Frank, C. W., Surface-Initiated Vapor Polymerization of Various α-Amino Acids. *Langmuir* **2003,** *19* (4), 1295-1303.

[13] Chang, Y.-C.; Frank, C. W., Vapor Deposition−Polymerization of α-Amino Acid N-Carboxy Anhydride on the Silicon(100) Native Oxide Surface. *Langmuir* **1998,** *14* (2), 326-334.

[14] Deng, X.; Friedmann, C.; Lahann, J., Bio-orthogonal "double-click" chemistry based on multifunctional coatings. *Angew Chem Int Ed Engl* **2011,** *50* (29), 6522-6.
